# SARS-CoV-2 Infects Hamster Testes

**DOI:** 10.3390/microorganisms9061318

**Published:** 2021-06-17

**Authors:** Rafael K. Campos, Vidyleison N. Camargos, Sasha R. Azar, Clint A. Haines, Eduardo J. Eyzaguirre, Shannan L. Rossi

**Affiliations:** 1 Department of Microbiology and Immunology, University of Texas Medical Branch, Galveston, TX 77555, USA; rkkroonc@utmb.edu (R.K.C.); cahaines@utmb.edu (C.A.H.); 2 Department of Pathology, University of Texas Medical Branch, Galveston, TX 77555-0609, USA; vinevesc@utmb.edu (V.N.C.); srazar@utmb.edu (S.R.A.); ejeyzagu@utmb.edu (E.J.E.); 3 Institute for Human Infection and Immunity, University of Texas Medical Branch, Galveston, TX 77555, USA

**Keywords:** SARS-CoV-2, testes, Sertoli, hamster, reproductive tract

## Abstract

The COVID-19 pandemic continues to affect millions of people worldwide. Although SARS-CoV-2 is a respiratory virus, there is growing concern that the disease could cause damage and pathology outside the lungs, including in the genital tract. Studies suggest that SARS-CoV-2 infection can damage the testes and reduce testosterone levels, but the underlying mechanisms are unknown and evidence of virus replication in testicular cells is lacking. We infected golden Syrian hamsters intranasally, a model for mild human COVID-19, and detected viral RNA in testes samples without histopathological changes up to one month post-infection. Using an ex vivo infection model, we detected SARS-CoV-2 replication in hamster testicular cells. Taken together, our data raise the possibility that testes damage observed in severe cases of COVID-19 could be partly explained by direct SARS-CoV-2 infection of the testicular cells.

## 1. Introduction

The COVID-19 pandemic is a global health emergency caused by the severe acute respiratory syndrome virus 2 (SARS-CoV-2), a virus from the genus *Betacoronavirus* and family *Coronaviridae* [1,2]. Human patient data suggest that SARS-CoV-2 may be able to infect several organs in addition to the lungs, including the heart, kidneys, and brain [3,4,5,6,7,8]. Infection of the male genital tract (MGT) has been reported for a variety of viruses, including filoviruses (Ebola and Marburg viruses), flaviviruses (Zika virus), and paramyxoviruses (mumps virus), with implications for sexual transmission, persistence, fertility, and testicular health [9,10,11,12,13,14,15,16]. The MGT may play a role in SARS-CoV-2 pathogenesis as COVID-19 has higher infection and fatality rates in men as compared to women [17], suggesting that the MGT may function as a site of virus replication or influence disease severity. It is becoming increasingly clear that COVID-19 affects testes [18], as evidenced by pain in the testicular area [19,20,21], ultrasound and histological analyses [22,23,24,25], reduced number of Leydig cells that produce androgen hormones [26,27], reduced testosterone levels [26,28], and the presence of testicular inflammation [22]. Spermatogenesis may also be affected [24,25], and SARS-CoV-2 RNA has been detected in the semen [29]. It is not clear, however, if testicular damage observed in certain severe COVID-19 cases is caused by direct SARS-CoV-2 infection, indirect inflammatory and oxidative stress, or a combination of these mechanisms.

SARS-CoV-2 has been shown to enter cells through one of two pathways, which require, in addition to its receptor angiotensin-converting enzyme 2 (ACE2), either cathepsins or transmembrane protease serine 2 (TMPRSS2) to efficiently complete its entry step [30,31,32,33]. Qi and colleagues analyzed expression of ACE2 and TMPRSS2 and found that cell types in the testes that express both proteins include spermatogonia, spermatogonia stem cells, peritubular myoid cells, and testes somatic cells [34]. Another study by Wang and colleagues found that ACE2 and TMPRSS2 are co-expressed in many cell types in the testes, including spermatogonia, spermatocytes, spermatids, somatic, Leydig, and Sertoli cells. Among these cells, high expression of ACE2 were found in spermatogonia, Sertoli, and Leydig cells, while high expression of TMPRSS2 were detected in spermatogonia and spermatids. Further, androgens positively modulate the expression of TMPRSS2 [35,36]. Another cellular receptor used by the virus, Cathepsin-L, is normally found in germ cells and Sertoli cells [37]. Taken together, these studies suggest that several cell types in the testes have the receptors required for entry of SARS-CoV-2.

Studies analyzing the levels of ACE2, TMPRSS2, and cathepsins suggest that the testes are potentially vulnerable to SARS-CoV-2 [25,34,37,38]. However, it is unknown whether SARS-CoV-2 can replicate and complete its life cycle in testicular cells. Autopsy data has shown viral antigen in testicular tissue [25], but there have been no in vitro or animal model data to directly measure infection and replication. Animal and cell culture models are important models to carry out these infections and generate data complementary to human studies, allowing researchers to control for confounding variables such as age, diet, environment, time of infection, and postmortem changes in the tissue. Among the known animal models for COVID-19, the golden Syrian hamster models mild cases of the disease, having disease signs comparable to humans that contract the disease but are able to recover without apparent sequelae. Using this model, we describe the detection of SARS-CoV-2 viral RNA in hamster testes in the absence of remarkable histopathological changes. Strikingly, we find that SARS-CoV-2 can infect and replicate in hamster testicular cells ex vivo, suggesting that the testes may be a site of transient replication in non-fatal COVID-19 cases.

## 2. Materials and Methods

### 2.1. Cells and Viruses Used

Vero E6 cells (American Type Culture Collection, Manassas, VA, USA, CRL-1586) were grown in Dulbecco’s minimal essential media (DMEM with L-glutamine and 4.5 g/L of D-glucose, without sodium pyruvate, Gibco, Thermo Fisher Scientific, Waltham, MA, USA) supplemented with 10% of fetal bovine serum (FBS, Atlanta Biologicals, Flowery Branch, GA, USA), 200 mg/mL streptomycin, and 200 U/mL penicillin (DMEM, Gibco, Thermo Fisher Scientific, Waltham, MA, USA). Cells were incubated at 37 °C with 5% CO_2_. Manipulations of SARS-CoV-2 were done under biosafety level 3 (BSL-3) conditions. The SARS-CoV-2 strain used was obtained from the World Reference Center for Emerging Viruses and Arboviruses (WRCEVA) at passage 4 (in Vero cells) and passaged once more in Vero E6 cells to produce a working stock. Virus was used to infect the Vero monolayer at a multiplicity of infection (MOI) of 0.001 and allowed to contact cells for 1 h in an incubator set to 37 °C with 5% of CO_2_, with rocking of the plate done every 15 min. The media was then removed and 20 mL of DMEM supplemented with 2% FBS and antibiotics were added to the flask. The cytopathic effect was observed via light microscopy (Olympus CK2, Tokyo, Japan) and the supernatant collected 3 days post infection (dpi), spun for 5 min at 3000× *g*, aliquoted, and stored at −80 °C.

### 2.2. Animal Infection

**Figure 1:**
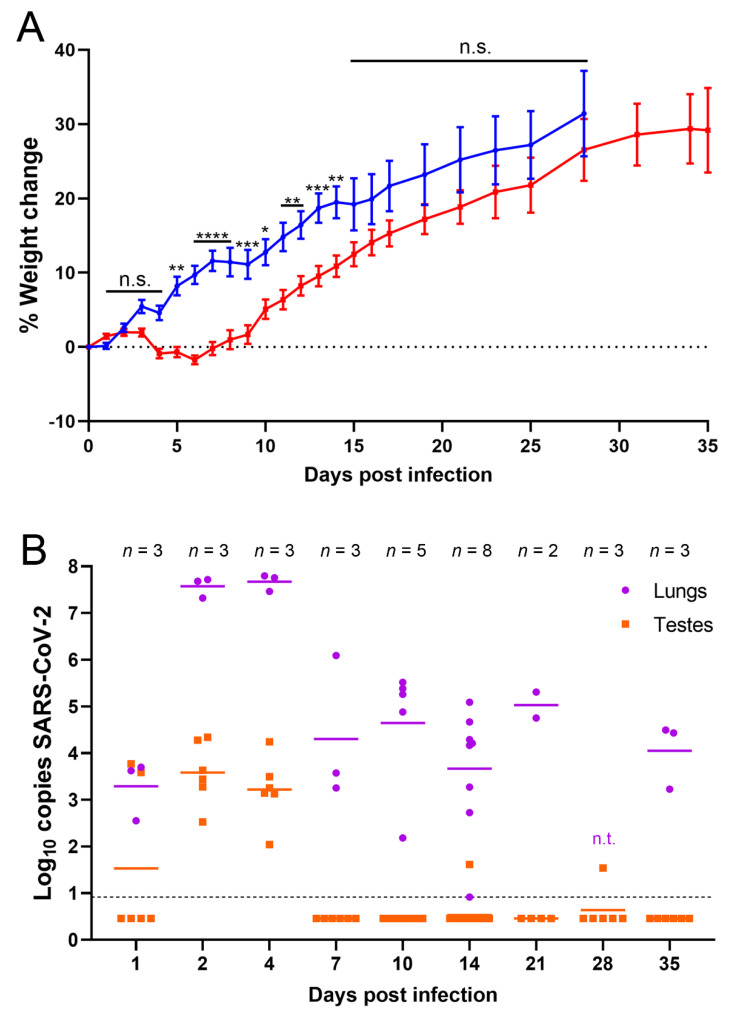
SARS-CoV-2 RNA was detected in testes of infected hamsters. (**A**) Percent weight change of infected (red, target dose 1 × 10^5^ PFU) and uninfected (blue, PBS) hamsters. Individual hamsters’ data are plotted and the line represents the average. Weight data is from one experiment out of three done. Error bars represent the standard error of the mean. Asterisks represent p values from statistical comparisons of infected vs uninfected groups at each timepoint: * *p* < 0.05, ** *p* < 0.01, *** *p* < 0.001, **** *p* < 0.0001. n.s. stands for not significative, *p* ≥ 0.05; (**B**) RT-qPCR results from homogenized lung (purple) and testes (orange). n.t. indicates samples that were not tested by RT-qPCR. Samples below the dashed line were undetectable after 40 cycles. Undetectable samples were represented as half of the lowest sample detected (0.46). Panel B shows data of three experiments combined.

Six- to eight-week-old male Syrian golden hamsters (*Mesocricetus auratus*, Charles River, Houston, TX, USA) were infected with 1 × 10^5^ plaque-forming units (PFU)/animal by intranasal inoculation of virus diluted in 20 µL of phosphate buffered saline (DPBS without calcium and magnesium, Gibco, Thermo Fisher Scientific, Waltham, MA, USA). The animal weights were taken daily up to 14 dpi and once every 3 days after that. Animal manipulations were done in accordance with Institutional Animal Care and Use Committee (IACUC) protocol number 2007076. Hamsters were euthanized on pre-established days after infection via CO_2_ asphyxiation followed by a secondary method (bilateral thoracotomy). No hamsters reached the pre-established humane endpoints during this study. The results shown are a combination of three independent cohorts. The backtiters of each cohort were 1.2 × 10^5^, 8.4 × 10^4^ (used for Figure 1A), and 7.2 × 10^4^ PFU/hamster.

### 2.3. Plaque Assays

Virus was quantified as previously described [39]. Briefly, Vero E6 cells were seeded in 6-well plates and serially diluted samples were adsorbed for 1 h then overlaid with 4 mL of a semisolid overlay consisting of minimum essential medium (MEM, Gibco, Thermo Fisher Scientific, Waltham, MA, USA) without phenol red, 0.8% agarose solution, 4% FBS, and antibiotics. The plates were incubated for 48 h at 37 °C at 5% of CO_2_ and then stained with 2 mL of a neutral red solution of 0.05% (Thermo Fisher Scientific, Waltham, MA, USA) for 6 h. The neutral red solution was then removed and plaques were visualized using a lightbox and counted.

### 2.4. Ex Vivo Testes Infection

Both testes of an uninfected, 7-week-old golden Syrian hamster were collected and cut into 3 equal-sized pieces. Each piece was added to a well of a 12-well plate and infected using 300 µL of stock virus containing 1.5 × 10^7^ PFU, similar to what has been described in other publications of ex vivo testes models [40,41]. The virus adsorption was allowed to proceed for 1 h, then the media were removed and the well was washed with 1 mL of PBS 4 times prior to incubation at 37 °C, 5% CO_2_ for 16, 48, or 72 h with Sertoli cell basal media (iX Cell Biotechnologies, San Diego, CA, USA, product code MD-0091B). As a control for immunofluorescence downstream analysis, Vero E6 cells were seeded in tissue culture slides (Corning, Corning, NY, USA) at 50,000 cells per chamber and grown as described before for 24 h. Cells were transferred to a BSL-3 laboratory before infection with SARS-CoV-2 at an MOI of 1 and fixed with 10% neutral buffered formalin (Thermo Fisher Scientific, Waltham, MA, USA).

### 2.5. Sertoli and Vero Cells Growth Curves

Vero E6 cells or human primary Sertoli (hpSertoli) cells (iX Cells Biotechnologies, San Diego, CA, USA, product code 10HU-149) were prepared as previously described [42], with some modifications. Briefly, cells were plated in 6-well plates at 4 × 10^5^ and 2 × 10^5^ cells/well, respectively, and infected the following day with an MOI of 10 of SARS-CoV-2 in 250 µL DMEM media supplemented with 2% of FBS and penicillin and streptomycin as above. The infection was allowed to proceed for 1 h in a 37 °C incubator with 5% of CO_2_. Cells were then washed with PBS four times and Sertoli cell basal media was completed to 2 mL before incubation under the same conditions as above for 48 h. An aliquot of 500 µL of the supernatant was collected at 5 min, 24 h, and 48 h post-infection (hpi), frozen at −80 °C, and used at a later date for titration via plaque assay as described above.

### 2.6. RT-qPCR

RNA of clarified tissue homogenates was extracted using Trizol LS reagent (Thermo Fisher Scientific, Waltham, MA, USA) and eluted in 30 µL of RNase-free water as previously described [39]. RT-qPCRs were performed on a StepOne (Thermo Fisher Scientific, Waltham, MA, USA) system using Taq Man RNA-to-CT 1-Step kits (Thermo Fisher Scientific, Waltham, MA, USA) according to the manufacturer’s conditions. Each reaction used 600 nM of forward primer (GTG AAA TGG TCA TGT GTG GCG G), 800 nM of reverse primer (CAA ATG TTA AAA ACA CTA TTA GCA TA), and 200 nM of probe (SUN-CAG GTG GAA CCT CAT CAG GAG ATG C; quenched with an internal ZEN and a 3′ Iowa Black) (IDT, Coralville, IA, USA, product code 10007063); these primers and probes were modified [43] to match the specific virus strain used in our studies. The RT-qPCR reaction conditions consisted of the following steps: 48 °C for 30 min, 95 °C for 10 min, 40 cycles of 95 °C for 15 s, and 58 °C for 1 min.

RNA of a stock virus sample (8 × 10^6^ PFU/mL) was extracted and diluted to generate a standard curve for absolute virus quantification. Stock RNA was quantified by a Qubit fluorometer (Thermo Fisher Scientific, Waltham, MA, USA) and the copy number was calculated based on the SARS-CoV-2 genome size (29,870 nt). The standard curve was run in every plate and the copy number of each sample was calculated using the standard curve values corresponding to their respective plate. All amplified samples were shown as log_10_ of the copy number per sample. The average sample weight collected from lungs was 0.4 g and for testes was 0.45 g. The RT-qPCR results of serum and blood described in the results are from sera samples collected from hamsters across two independent experiments (2 dpi N = 3; 4 dpi N = 3; 7 dpi N = 2; 10 dpi N = 2; 14 dpi N = 5; 28 dpi N = 3) and blood samples collected in a third independent experiment (1 dpi N =3; 7 dpi N = 1; 10 dpi N = 3; 14 dpi N = 3; 21 dpi N = 2; 35 dpi N = 3). Undetectable samples were plotted in Figure 1B as half of the sample with the lowest detectable copy number (0.46).

### 2.7. Histology

Tissue samples were collected and immediately added to 10% neutral buffered formalin (Thermo Fisher Scientific, Waltham, MA, USA) for 2 days. The formalin was changed and tissue fixed for an additional 7 days. SARS-CoV-2 ex vivo-infected testes were also fixed using this same method. Samples were then transferred to 95% ethanol until paraffin embedding was performed by UTMB’s Anatomic Pathology Laboratory core facility. Five micrometer sections were used for hematoxylin-eosin (H&E) staining by the core facility for immunofluorescence analyses. A blinded board-certified pathologist read the H&E slides.

### 2.8. Immunofluorescence Assay

Immunofluorescence assays were conducted with a modified protocol from a previous study [44]. Sections of hamster testes on slides were baked at 65 °C overnight and treated with 0.05 mg/mL proteinase K for 20 min at 37 °C. SARS-CoV-2-infected Vero cells were incubated with Triton-X 0.1% for 10 min at room temperature. Both tissue sections and cell monolayers were blocked with 3% FBS in PBS for 15 min and stained with the mouse-produced anti-dsRNA antibody J2 1:500 (Scicons, Susteren, The Netherlands, product number: 10010200) at 4 °C overnight. Anti-mouse IgG secondary antibody AlexaFluor 594 (Thermo Fisher, Waltham, MA, USA, product code A11005) was added onto sections at 1:1000 dilution, incubated for 45 min at 37 °C, and slides were mounted with ProLong Gold Antifade Mountant with DAPI (Thermo Fisher, Waltham, MA, USA, product code P36931). Vero cells were visualized under an inverted fluorescence microscope (Olympus-IX73, Tokyo, Japan). Testes sections were imaged via confocal microscopy. Large area tissue overview images were obtained using tile scan and stitching of low-resolution images (0.8× scan zoom, 126 × 126 pixels, 10% overlap) with a Zeiss 880 laser scanning microscope (Jena, Germany) and a Plan-Apochromat 63×/1.4 oil DIC M27 objective lens. DAPI and Alexa Fluor 594 fluorophores were excited with 405 nm and 561 nm laser lines, respectively, and fluorescence emissions were detected in the 410–507 nm and 591–669 nm wavelength bands, respectively. The pixel depth was 8-bit and the pinhole was set to 1.59 airy units (AU) for DAPI and 1.16 AU for Alexa Fluor 594. Image tiles were stitched in Zeiss ZEN 2.3 SP1 software (black, version 14). Z-stacks and individual snapshots of regions of interest chosen from overviews were captured by the same software (1.0× scan zoom, 1912 × 1912 pixels). Images captured from infected and uninfected tissues both had their thresholds set to the same levels to remove background. All post-processing adjustments were applied equally to all images from both uninfected and infected tissues.

### 2.9. Quantification and Statistical Analyses

Weight data from SARS-CoV-2-inoculated hamsters at each timepoint were compared to that of control PBS-inoculated hamsters at the corresponding timepoint via the mixed-effects model for repeated measures analysis. The Sidak test was the posthoc test used to correct for comparisons. Virus titers were transformed to log_10_ PFU/mL prior to analysis, compared via one-way ANOVA, and P-values lower than 0.05 were considered significant. Statistical analyses were conducted using the GraphPad Prism software version 9.

## 3. Results and Discussion

### 3.1. Golden Syrian Hamsters Infected with SARS-CoV-2 Show Signs of Mild Disease

Previous studies suggested that SARS-CoV-2 infection can damage the male reproductive tract either through direct infection or indirectly through inflammation and oxidative stress. To determine the ability of SARS-CoV-2 to infect the testes in vivo, golden Syrian hamsters were used to model mild, non-lethal COVID-19 infection [45]. Across three independent experiments, hamsters were infected with approximately 1 × 10^5^ PFU intranasally and monitored daily for signs of disease and weight loss. The observed signs of disease were slight lethargy and ruffled coat in the SARS-CoV-2-infected hamsters. No signs of severe disease were observed. Infected hamsters lost weight or gained less weight during the first week and a half following infection compared to control hamsters intranasally inoculated with PBS (Figure 1A). Infected hamsters were significantly lower in weight than control hamsters from 5 to 14 dpi, with the largest differential in weight occurring between 6 and 8 dpi, after which their weights started to recover (Figure 1A).

### 3.2. SARS-CoV-2 RNA Was Detected in Testes Tissue without Histopathological Changes

To determine if SARS-CoV-2 was able to infect and damage the testes following an intranasal infection, testes of infected hamsters were harvested at 1, 2, 4, 7, 10, 14, 21, 28, and 35 dpi to detect viral RNA and characterize histological changes. Lungs, sera, and blood were collected as controls at these timepoints to confirm infection and rule out the possibility of viral load in organs from viremia. RT-qPCRs for SARS-CoV-2 RNA were performed from homogenized tissue supernatants. As expected, all lungs tested were positive for viral RNA via RT-qPCR, indicating that all animals became infected after intranasal inoculation with SARS-CoV-2; on average 3.3 log_10_ copies per sample at 1 dpi and at 2 and 4 dpi the RNA levels peaked at 7.6 and 7.7 log_10_ copies per sample, respectively (Figure 1B). At 7 dpi and later timepoints, there was a reduction in virus RNA in the lungs with an average of 5 log_10_ or fewer copies per sample (Figure 1B). This correlated with the timepoint at which hamsters start recovering weight (Figure 1A). In contrast to the lung samples, viral RNA was only detected in one sample of blood (at 1 dpi, which had 2.9 log_10_ copies of viral RNA) and 2 samples of sera (at 4 and 7 dpi, which had 1.4 log_10_ and 1.7 log_10_ copies of viral RNA, respectively). Viral RNA was detected in testes homogenates at 1 dpi from one of three animals measuring 3.6 log_10_ copies (left) and 3.8 log_10_ copies (right) (Figure 1B). Importantly, all samples from 2 and 4 dpi were also positive and contained on average 3.6 and 3.2 log_10_ copies of viral RNA, respectively (Figure 1B). Two right testes samples were positive with low copies of viral RNA at later timepoints: one at 14 dpi (1.6 log_10_ copies of viral RNA) and a second at 28 dpi (1.5 log_10_ copies of viral RNA) (Figure 1B). These data suggest that SARS-CoV-2 is capable of infecting the testes soon after intranasal infection and viral RNA is detectable within the first week of infection but is resolved in most hamsters soon thereafter.

To determine whether infection affected testicular health, testes collected at 14, 21, and 28 dpi, as well as controls collected at 28 dpi, were H&E stained and analyzed blindly. No remarkable pathology was noted in the seminiferous tubules or interstitium at these timepoints, suggesting that the SARS-CoV-2 RNA present in the testes does not correlate with significant damage (Figure S1). This is in contrast with some of the publications analyzing human testes postmortem [25] and may be explained by the fact that infection of hamsters has more similarities with mild human cases of COVID-19 than with the severe cases analyzed in postmortem studies.

Testes have been classified as an immune-privileged organ, as are the eyes, brain, and hair follicles, and understanding the interaction between a pathogen and the immune system in this type of organ is critical [46]. Oftentimes the immune system activity results in significant damage, which can lead to testes dysfunction and impact reproductive health. Macrophages play a critical role in the development of uncontrolled testicular infection [11,47] and were present in the testes of COVID-19 autopsy patients [24,25]. In this hamster model, no remarkable pathology was observed, the blood–testis barrier (BTB) appeared intact and there was no increase in immune cells within the tissue. It is possible that this low level of infection was insufficient to trigger an immune response. Whether testicular damage is virus-mediated, immune-mediated, or both is currently unknown.

SARS-CoV-2 was likely unable to initiate a significant productive infection in vivo in these previously healthy (no pre-existing condition) hamsters. The testes of infected and uninfected hamsters appeared unremarkable, the BTB was intact between Sertoli cells, Leydig cell cluster frequency was not reduced, and spermatogenesis was unaffected. These hamsters were young, sexually mature adults—an age group associated with “mild” COVID-19 disease in humans. Severe disease is associated with age and pre-existing conditions like hypertension and diabetes, and, interestingly, all these conditions are linked to significant disruptions in the BTB and overall testicular/sperm health and function [48,49,50]. Most of the autopsies where infection and damage to the testes and sperm were noted had these co-morbidities [25].

### 3.3. SARS-CoV-2 Can Infect Cells of the Testicular Tissue Ex Vivo

Although we detected SARS-CoV-2 RNA in the testes of infected hamsters, presence of viral RNA does not equate to the presence of viable virus and does not address whether the virus is able to replicate in testicular cells. In fact, the ratio of RNA to infectious particles is variable and depends on many factors, including the timepoint at which the samples were collected [51].

**Figure 2:**
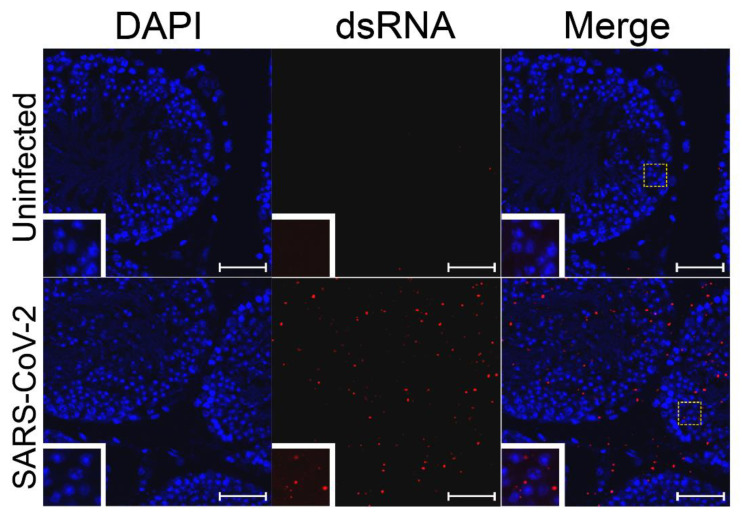
SARS-CoV-2 dsRNA in ex vivo-infected testes. Ex vivo testicular tissue was fixed and sectioned 24 hpi. Sections were stained for dsRNA (J2, red) and for cell nuclei (DAPI, blue). The white dashed boxes show the region of the figure from which the inset was magnified. White bars indicate a length of 5 µm.

To test whether SARS-CoV-2 is able to replicate in cells of the testes, testes from uninfected hamsters were infected ex vivo with 1 × 10^7^ PFU of SARS-CoV-2 strain USA_WA1/2020 for 24 h. The pieces of testes were then fixed and embedded in paraffin blocks. The blocks with uninfected control or ex vivo infected testes were sectioned and subjected to immunofluorescence using J2 antibodies against dsRNA, which detects the RNA replication step of the virus when the positive and negative strains are annealed to each other. As a positive control, SARS-CoV-2-susceptible Vero cells were used to confirm infection (Figure S2). Staining of dsRNA was observed in many infected cells inside of the seminiferous tubules and on the interstitium, indicating replication of SARS-CoV-2 RNA in different testicular cell types (Figure 2 and Supplemental Video).

**Figure 3:**
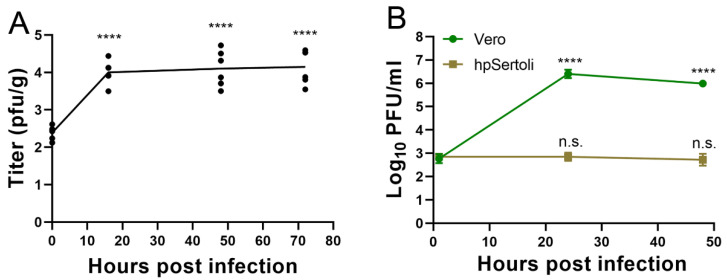
SARS-CoV-2 replicates in testicular cells but not hpSertoli cells. (**A**) Ex vivo infection of testes with SARS-CoV-2. Symbols show each well with a piece of testes, the line shows the average. **** denotes a *p* < 0.0001 in a one-way ANOVA test of each timepoint compared to the initial timepoint. Representative of two independent experiments. (**B**) Growth curve in Vero cells or hpSertoli cells at an MOI of 10. Limit of detection is 4 PFU/mL. **** denotes p in a one-way ANOVA test of each timepoint compared to the initial timepoint. Representative of two independent studies. Error bars of (**A**) and (**B**) represent standard deviation. n.s. stands for not significative, *p* ≥ 0.05.

Using ex vivo infection of pieces of testes under the same conditions, a growth curve was performed to quantify virus production from the pieces of testes. SARS-CoV-2 was able to productively infect testes in this ex vivo model, as evidenced by increasing titers from 2.4 log_10_ PFU/gram, to 4.1 log_10_ PFU/gram at 16 hpi (Figure 3A). Titers remained constant thereafter, suggesting an equilibrium was reached between virus replication and degradation (Figure 3A).

Both in vivo and ex vivo data from this study suggest that SARS-CoV-2 is able to infect the testes, although productive infection was only found in the ex vivo model. This lack of infectious particles in vivo may be due to several factors, including (1) intranasal infection in this hamster model is non-lethal and represents “mild” human disease and is resolved by the immune system, (2) the cell types that support productive infection were not reached by the virus in the testes of a healthy animal, (3) severe infection and pathology are associated with pre-existing conditions known to affect testicular health, and (4) infection may be influenced by the strain and/or passage history of SARS-CoV-2.

The ex vivo data indicate that the testes could be a site of virus amplification. The virus could theoretically be transported to the epididymis via the excurrent duct system and end up in the semen resulting in sexual transmission. However, it is unknown if SARS-CoV-2 can replicate in the epididymis; the virus has not been isolated from semen to date and presence of the viral RNA in human semen has only observed in seven patients across two studies [29,52] and therefore it is thought to be a rare occurrence.

Testes contain many specialized cell types. Sertoli cells form tight junctions between each other to form the BTB, whose functions include preventing the immune system and pathogens from interacting with developing sperm. Sertoli cells are also a primary site of Zika [53] and Marburg [11] infections. To determine if ACE2-expressing Sertoli cells are susceptible to SARS-CoV-2 infection, human primary Sertoli cells were exposed to the virus at a multiplicity of infection of 10 and the supernatant was titrated via plaque assay. Vero cells served as a susceptible cell control. In Vero cells, SARS-CoV-2 replicates to titers upwards of 6 log_10_ PFU/mL (Figure 3B). In contrast, SARS-CoV-2 titers remained stable in hpSertoli cells, suggesting that these cells may support low levels of SARS-CoV-2 infection (Figure 3B). As the starting amount of virus was 2.9 log_10_ PFU/mL after the cells were washed with PBS, and these titers remained constant, this indicates there is a small amount of replication, since there is an equilibrium between production and degradation, as degradation of SARS-CoV-2 in DMEM has been shown to be 6.5 log_10_ in 2 days (Figure 3B) [54]. Therefore, it is likely that another cell type in the testes plays a critical role in virus amplification during in vivo infection.

The virus strain used for these studies was USA_WA1/2020 (MN985325.1) [55], an isolate made from the first human COVID-19 case in the United States in January 2020 [56]. SARS-CoV-2 has since evolved and numerous variants with increased transmissibility and pathogenicity have emerged in different parts of the world. It is possible that variants of concern, including the B1.351 (Beta) and P.1 (Gamma) variants, could have an increased host range, making murine studies feasible without the need to adapt the virus [57]. The testicular pathogenicity of these variants remains unknown.

## 4. Conclusions

As of the writing of this article, the COVID-19 pandemic continues to affect millions of people worldwide with limited signs of abating. Although primarily a respiratory infection [58], the systemic effects of SARS-CoV-2 infection have been observed and the full scope of pathogenesis and development of sequalae is still under investigation [59,60,61,62]. Here, we describe the possibility for SARS-CoV-2 to infect the testes. The real burden of disease on the MGT is still unknown but the role of this organ system in viral infections, including Zika and Ebola/Marburg viruses, is starting to be fully appreciated. Understanding the complete pathogenesis of SARS-CoV-2 is critical to the long-term health of the millions of COVID-19 patients, including “long-haulers” [63].

Our study detected SARS-CoV-2 RNA in the testes of golden Syrian hamsters infected intranasally. Viral RNA was mostly detected during the first week post-infection and we did not observe prominent histopathological damage. Hamsters’ testes exposed to SARS-CoV-2 via ex vivo infection were susceptible to infection as evidenced by increasing virus titers in the media and dsRNA detection in the seminiferous tubules and interstitium. Additionally, hpSertoli cells displayed low levels of viral replication after exposure to SARS-CoV-2. Taken together, our data suggest that testicular damage observed in severe COVID-19 cases could be partly due to direct SARS-CoV-2 infection of the testicular cells. Using animal models to understand the role of pre-conditions associated with severe COVID-19 on testicular infection, differential damage caused by SARS-CoV-2 variants, and whether SARS-CoV-2 can be sexually transmitted are crucial steps for studies.

## Data Availability

The data presented in this study may be available on request from the corresponding author.

## Supplementary Materials

The following are available online at https://www.mdpi.com/article/10.3390/microorganisms9061318/s1, Figure S1: SARS-CoV-2 intranasal infection of golden Syrian hamsters does not cause histopathological changes in their testes, Figure S2: SARS-CoV-2 dsRNA in infected Vero cells, Video S1: Z-stack of SARS-CoV-2 dsRNA in cells of ex vivo infected testes.

## References

[1] Andersen K.G., Rambaut A., Lipkin W.I., Holmes E.C., Garry R.F. (2020). The proximal origin of SARS-CoV-2. Nat. Med..

[2] Zhu N., Zhang D., Wang W., Li X., Yang B., Song J., Zhao X., Huang B., Shi W., Lu R. (2020). A Novel Coronavirus from Patients with Pneumonia in China, 2019. N. Engl. J. Med..

[3] Bailey A.L., Dmytrenko O., Greenberg L., Bredemeyer A.L., Ma P., Liu J., Penna V., Winkler E.S., Sviben S., Brooks E. (2021). SARS-CoV-2 Infects Human Engineered Heart Tissues and Models COVID-19 Myocarditis. JACC Basic Transl. Sci..

[4] Lindner D., Fitzek A., Brauninger H., Aleshcheva G., Edler C., Meissner K., Scherschel K., Kirchhof P., Escher F., Schultheiss H.P. (2020). Association of Cardiac Infection with SARS-CoV-2 in Confirmed COVID-19 Autopsy Cases. JAMA Cardiol..

[5] Meinhardt J., Radke J., Dittmayer C., Franz J., Thomas C., Mothes R., Laue M., Schneider J., Brünink S., Greuel S. (2021). Olfactory transmucosal SARS-CoV-2 invasion as a port of central nervous system entry in individuals with COVID-19. Nat. Neurosci..

[6] Sahoo P.R., Sahu M., Surapaneni P.S., Maiti A., Vankamamidi R., Panda N., Biswal R.N. (2021). Evolution of olfactory and gustatory dysfunctions in COVID-19 patients in India. Eur. Arch. Otorhinolaryngol..

[7] Diao B., Wang C., Wang R., Feng Z., Zhang J., Yang H., Tan Y., Wang H., Wang C., Liu L. (2021). Human kidney is a target for novel severe acute respiratory syndrome coronavirus 2 infection. Nat. Commun..

[8] Jiao L., Yang Y., Yu W., Zhao Y., Long H., Gao J., Ding K., Ma C., Li J., Zhao S. (2021). The olfactory route is a potential way for SARS-CoV-2 to invade the central nervous system of rhesus monkeys. Signal Transduct. Target. Ther..

[9] Deen G.F., Broutet N., Xu W., Knust B., Sesay F.R., McDonald S.L., Ervin E., Marrinan J.E., Gaillard P., Habib N. (2017). Ebola RNA Persistence in Semen of Ebola Virus Disease Survivors-Final Report. N. Engl. J. Med..

[10] Thorson A.E., Deen G.F., Bernstein K.T., Liu W.J., Yamba F., Habib N., Sesay F.R., Gaillard P., Massaquoi T.A., McDonald S.L.R. (2021). Persistence of Ebola virus in semen among Ebola virus disease survivors in Sierra Leone: A cohort study of frequency, duration, and risk factors. PLoS Med..

[11] Coffin K.M., Liu J., Warren T.K., Blancett C.D., Kuehl K.A., Nichols D.K., Bearss J.J., Schellhase C.W., Retterer C.J., Weidner J.M. (2018). Persistent Marburg Virus Infection in the Testes of Nonhuman Primate Survivors. Cell Host Microbe.

[12] Rogstad K.E., Tunbridge A. (2015). Ebola virus as a sexually transmitted infection. Curr. Opin. Infect. Dis..

[13] Counotte M.J., Kim C.R., Wang J., Bernstein K., Deal C.D., Broutet N.J.N., Low N. (2018). Sexual transmission of Zika virus and other flaviviruses: A living systematic review. PLoS Med..

[14] Mead P.S., Duggal N.K., Hook S.A., DeLorey M., Fischer M., McGuire D.O., Becksted H., Max R.J., Anishchenko M., Schwartz A.M. (2018). Zika Virus Shedding in Semen of Symptomatic Infected Men. N. Engl. J. Med..

[15] Dejucq N., Jegou B. (2001). Viruses in the mammalian male genital tract and their effects on the reproductive system. Microbiol. Mol. Biol. Rev..

[16] Wu H., Wang F., Tang D., Han D. (2021). Mumps Orchitis: Clinical Aspects and Mechanisms. Front. Immunol..

[17] Meng Y., Wu P., Lu W., Liu K., Ma K., Huang L., Cai J., Zhang H., Qin Y., Sun H. (2020). Sex-specific clinical characteristics and prognosis of coronavirus disease-19 infection in Wuhan, China: A retrospective study of 168 severe patients. PLoS Pathog..

[18] Chen F., Lou D. (2020). Rising Concern on Damaged Testis of COVID-19 Patients. Urology.

[19] Gagliardi L., Bertacca C., Centenari C., Merusi I., Parolo E., Ragazzo V., Tarabella V. (2020). Orchiepididymitis in a Boy with COVID-19. Pediatr. Infect. Dis. J..

[20] Özveri H., Eren M.T., Kırışoğlu C.E., Sarıgüzel N. (2020). Atypical presentation of SARS-CoV-2 infection in male genitalia. Urol. Case Rep..

[21] Ediz C., Tavukcu H.H., Akan S., Kizilkan Y.E., Alcin A., Oz K., Yilmaz O. (2021). Is there any association of COVID-19 with testicular pain and epididymo-orchitis?. Int. J. Clin. Pract..

[22] Yang M., Chen S., Huang B., Zhong J.-M., Su H., Chen Y.-J., Cao Q., Ma L., He J., Li X.-F. (2020). Pathological Findings in the Testes of COVID-19 Patients: Clinical Implications. Eur. Urol. Focus.

[23] Flaifel A., Guzzetta M., Occidental M., Najari B.B., Melamed J., Thomas K.M., Deng F.-M. (2021). Testicular Changes Associated with Severe Acute Respiratory Syndrome Coronavirus 2 (SARS-CoV-2). Arch. Pathol. Lab. Med..

[24] Li H., Xiao X., Zhang J., Zafar M.I., Wu C., Long Y., Lu W., Pan F., Meng T., Zhao K. (2020). Impaired spermatogenesis in COVID-19 patients. EClinicalMedicine.

[25] Ma X., Guan C., Chen R., Wang Y., Feng S., Wang R., Qu G., Zhao S., Wang F., Wang X. (2021). Pathological and molecular examinations of postmortem testis biopsies reveal SARS-CoV-2 infection in the testis and spermatogenesis damage in COVID-19 patients. Cell. Mol. Immunol..

[26] Ma L., Xie W., Li D., Shi L., Ye G., Mao Y., Xiong Y., Sun H., Zheng F., Chen Z. (2021). Evaluation of sex-related hormones and semen characteristics in reproductive-aged male COVID-19 patients. J. Med. Virol..

[27] Selvaraj K., Ravichandran S., Krishnan S., Radhakrishnan R.K., Manickam N., Kandasamy M. (2021). Testicular Atrophy and Hypothalamic Pathology in COVID-19: Possibility of the Incidence of Male Infertility and HPG Axis Abnormalities. Reprod. Sci..

[28] Temiz M.Z., Dincer M.M., Hacibey I., Yazar R.O., Celik C., Kucuk S.H., Alkurt G., Doganay L., Yuruk E., Muslumanoglu A.Y. (2021). Investigation of SARS-CoV-2 in semen samples and the effects of COVID-19 on male sexual health by using semen analysis and serum male hormone profile: A cross-sectional, pilot study. Andrologia.

[29] Li D., Jin M., Bao P., Zhao W., Zhang S. (2020). Clinical Characteristics and Results of Semen Tests among Men with Coronavirus Disease 2019. JAMA Netw. Open.

[30] Ashhurst A.S., Tang A.H., Fajtova P., Yoon M., Aggarwal A., Stoye A., Larance M., Beretta L., Drelich A., Skinner D. (2020). Potent in vitro anti-SARS-CoV-2 activity by gallinamide A and analogues via inhibition of cathepsin L.. bioRxiv.

[31] Bollavaram K., Leeman T.H., Lee M.W., Kulkarni A., Upshaw S.G., Yang J., Song H., Platt M.O. (2021). Multiple sites on SARS-CoV-2 spike protein are susceptible to proteolysis by cathepsins B, K, L, S, and V. Protein Sci..

[32] Hoffmann M., Kleine-Weber H., Schroeder S., Krüger N., Herrler T., Erichsen S., Schiergens T.S., Herrler G., Wu N.-H., Nitsche A. (2020). SARS-CoV-2 Cell Entry Depends on ACE2 and TMPRSS2 and Is Blocked by a Clinically Proven Protease Inhibitor. Cell.

[33] Prasad K., Ahamad S., Kanipakam H., Gupta D., Kumar V. (2021). Simultaneous Inhibition of SARS-CoV-2 Entry Pathways by Cyclosporine. ACS Chem. Neurosci..

[34] Qi J., Zhou Y., Hua J., Zhang L., Bian J., Liu B., Zhao Z., Jin S. (2021). The scRNA-seq Expression Profiling of the Receptor ACE2 and the Cellular Protease TMPRSS2 Reveals Human Organs Susceptible to SARS-CoV-2 Infection. Int. J. Environ. Res. Public Health.

[35] Lin B., Ferguson C., White J.T., Wang S., Vessella R., True L.D., Hood L., Nelson P.S. (1999). Prostate-localized and androgen-regulated expression of the membrane-bound serine protease TMPRSS2. Cancer Res..

[36] Jacquinet E., Rao N.V., Rao G.V., Zhengming W., Albertine K.H., Hoidal J.R. (2001). Cloning and characterization of the cDNA and gene for human epitheliasin. JBIC J. Biol. Inorg. Chem..

[37] Gye M.C., Kim S.T. (2004). Expression of cathepsin L in human testis under diverse infertility conditions. Arch. Androl..

[38] Wang Z., Xu X. (2020). scRNA-seq Profiling of Human Testes Reveals the Presence of the ACE2 Receptor, a Target for SARS-CoV-2 Infection in Spermatogonia, Leydig and Sertoli Cells. Cells.

[39] Plante J.A., Liu Y., Liu J., Xia H., Johnson B.A., Lokugamage K.G., Zhang X., Muruato A.E., Zou J., Fontes-Garfias C.R. (2020). Spike mutation D614G alters SARS-CoV-2 fitness. Nature.

[40] Matusali G., Houzet L., Satie A.-P., Mahé D., Aubry F., Couderc T., Frouard J., Bourgeau S., Bensalah K., Lavoué S. (2018). Zika virus infects human testicular tissue and germ cells. J. Clin. Investig..

[41] Roulet V., Denis H., Staub C., Le Tortorec A., Delaleu B., Satie A., Patard J., Jégou B., Dejucq-Rainsford N. (2006). Human testis in organotypic culture: Application for basic or clinical research. Hum. Reprod..

[42] Kumar A., Jovel J., Lopez-Orozco J., Limonta D., Airo A.M., Hou S., Stryapunina I., Fibke C., Moore R.B., Hobman T.C. (2018). Human Sertoli cells support high levels of Zika virus replication and persistence. Sci. Rep..

[43] Corman V.M., Landt O., Kaiser M., Molenkamp R., Meijer A., Chu D.K., Bleicker T., Brünink S., Schneider J., Schmidt M.L. (2020). Detection of 2019 novel coronavirus (2019-nCoV) by real-time RT-PCR. Eurosurveillance.

[44] Jacob F., Pather S.R., Huang W.-K., Zhang F., Wong S.Z.H., Zhou H., Cubitt B., Fan W., Chen C.Z., Xu M. (2020). Human Pluripotent Stem Cell-Derived Neural Cells and Brain Organoids Reveal SARS-CoV-2 Neurotropism Predominates in Choroid Plexus Epithelium. Cell Stem Cell.

[45] Imai M., Iwatsuki-Horimoto K., Hatta M., Loeber S., Halfmann P.J., Nakajima N., Watanabe T., Ujie M., Takahashi K., Ito M. (2020). Syrian hamsters as a small animal model for SARS-CoV-2 infection and countermeasure development. Proc. Natl. Acad. Sci. USA.

[46] Zhao S., Zhu W., Xue S., Han D. (2014). Testicular defense systems: Immune privilege and innate immunity. Cell. Mol. Immunol..

[47] Tsetsarkin K.A., Acklin J., Liu G., Kenney H., Teterina N.L., Pletnev A.G., Lim J.K. (2020). Zika virus tropism during early infection of the testicular interstitium and its role in viral pathogenesis in the testes. PLoS Pathog..

[48] Levy S., Serre V., Hermo L., Robaire B. (1999). The effects of aging on the seminiferous epithelium and the blood-testis barrier of the Brown Norway rat. J. Androl..

[49] Alves M.G., Martins A.D., Cavaco J.E., Socorro S., Oliveira P.F. (2013). Diabetes, insulin-mediated glucose metabolism and Sertoli/blood-testis barrier function. Tissue Barriers.

[50] Colli L.G., Belardin L.B., Echem C., Akamine E., Antoniassi M., Andretta R.R., Mathias L.S., Rodrigues S.F.D.P., Bertolla R.P., De Carvalho M.H.C. (2019). Systemic arterial hypertension leads to decreased semen quality and alterations in the testicular microcirculation in rats. Sci. Rep..

[51] Sia S.F., Yan L.-M., Chin A.W.H., Fung K., Choy K.-T., Wong A.Y.L., Kaewpreedee P., Perera R.A.P.M., Poon L.L.M., Nicholls J.M. (2020). Pathogenesis and transmission of SARS-CoV-2 in golden hamsters. Nat. Cell Biol..

[52] Machado B., Barra G.B., Scherzer N., Massey J., dos Santos Luz H., Jacomo R.H., Rita T.H.S., Davis R. (2021). Presence of SARS-CoV-2 RNA in Semen—Cohort Study in the United States COVID-19 Positive Patients. Infect. Dis. Rep..

[53] Govero J., Esakky P., Scheaffer S.M., Fernandez E.P.-A., Drury A., Platt D.J., Gorman M.J., Richner J., Caine E.A., Salazar V. (2016). Zika virus infection damages the testes in mice. Nat. Cell Biol..

[54] Chin A.W.H., Chu J.T.S., Perera M.R.A., Hui K.P.Y., Yen H.L., Chan M.C.W., Peiris M., Poon L.L.M. (2020). Stability of SARS-CoV-2 in different environmental conditions. Lancet Microbe.

[55] Harcourt J., Tamin A., Lu X., Kamili S., Sakthivel S.K., Murray J., Queen K., Tao Y., Paden C.R., Zhang J. (2020). Isolation and characterization of SARS-CoV-2 from the first US COVID-19 patient. bioRxiv.

[56] Holshue M.L., DeBolt C., Lindquist S., Lofy K.H., Wiesman J., Bruce H., Spitters C., Ericson K., Wilkerson S., Tural A. (2020). First Case of 2019 Novel Coronavirus in the United States. N. Engl. J. Med..

[57] Montagutelli X., Prot M., Levillayer L., Salazar E.B., Jouvion G., Conquet L., Donati F., Albert M., Gambaro F., Behillil S. (2021). The B1.351 and P.1 variants extend SARS-CoV-2 host range to mice. bioRxiv.

[58] Huang C., Wang Y., Li X., Ren L., Zhao J., Hu Y., Zhang L., Fan G., Xu J., Gu X. (2020). Clinical features of patients infected with 2019 novel coronavirus in Wuhan, China. Lancet.

[59] Carvalho T. (2020). Extrapulmonary SARS-CoV-2 manifestations. Nat. Med..

[60] Mehta O.P., Bhandari P., Raut A., Kacimi S.E.O., Huy N.T. (2021). Coronavirus Disease (COVID-19): Comprehensive Review of Clinical Presentation. Front. Public Health.

[61] Nathan N., Prevost B., Sileo C., Richard N., Berdah L., Thouvenin G., Aubertin G., LeCarpentier T., Schnuriger A., Jegard J. (2020). The Wide Spectrum of COVID-19 Clinical Presentation in Children. J. Clin. Med..

[62] Vodnar D.-C., Mitrea L., Teleky B.-E., Szabo K., Călinoiu L.-F., Nemeş S.-A., Martău G.-A. (2020). Coronavirus Disease (COVID-19) Caused by (SARS-CoV-2) Infections: A Real Challenge for Human Gut Microbiota. Front. Cell. Infect. Microbiol..

[63] Nalbandian A., Sehgal K., Gupta A., Madhavan M.V., McGroder C., Stevens J.S., Cook J.R., Nordvig A.S., Shalev D., Sehrawat T.S. (2021). Post-acute COVID-19 syndrome. Nat. Med..

