# Supplementary material for: SARS-CoV-2 Infects Hamster Testes
Source: Microorganisms. 2021 Jun 17;9(6):1318. doi: 10.3390/microorganisms9061318 (PMC8235703; doi:10.3390/microorganisms9061318)
Supplement: Supplementary file 1 [file microorganisms-09-01318-s001.zip › Supplemental_figures0508.pdf]

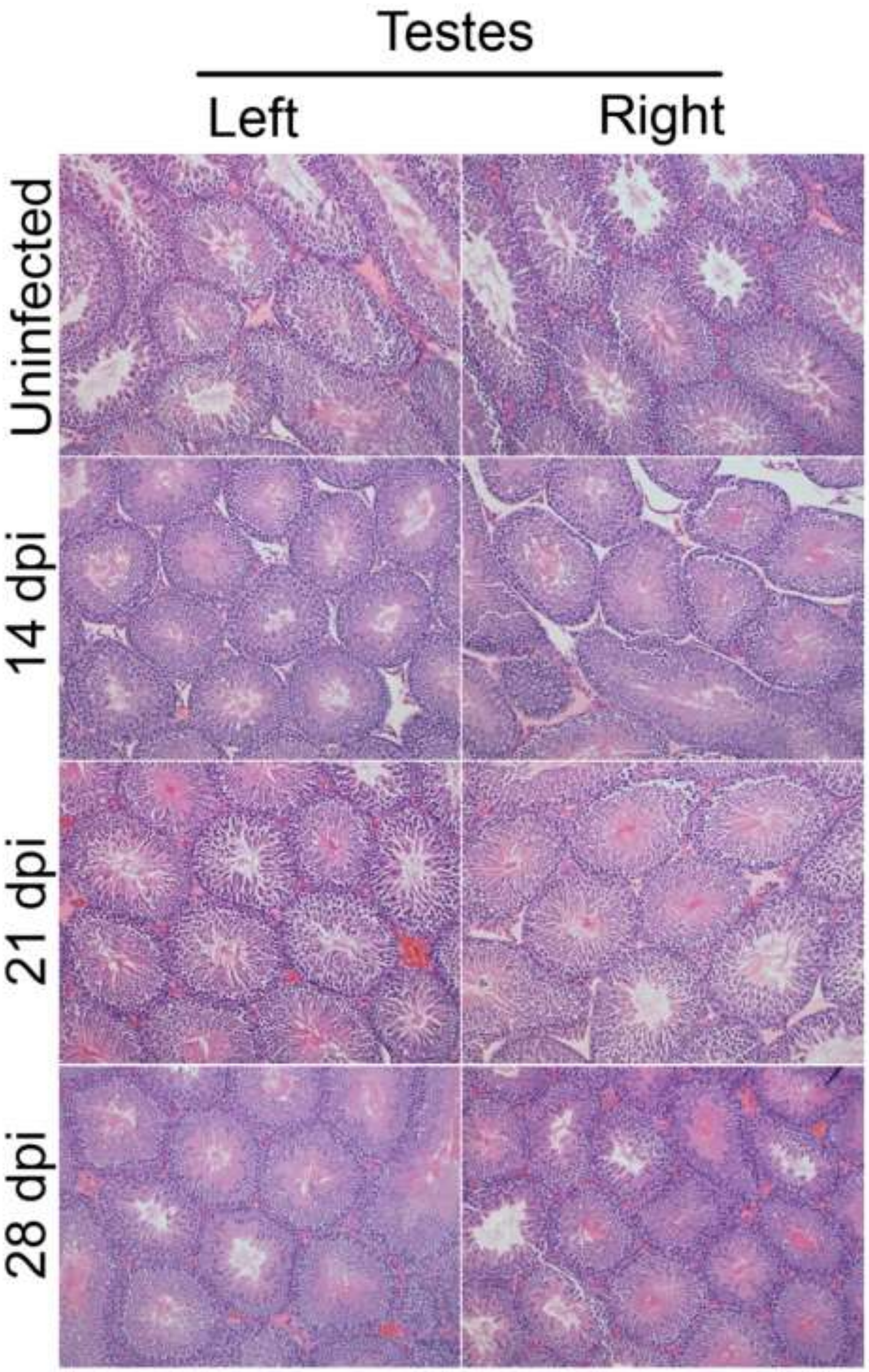

Figure S1. SARS-CoV-2 intranasal infection of golden Syrian hamsters does not cause histopathological changes in their testes. Representative H&E sections of formalin-fixed hamster testes (left and right) collected on days 14, 21 and 28 days post infection. The uninfected testes shown were collected 28 days post inoculation with PBS. Slides were visualized with a total magnification of 100 times.

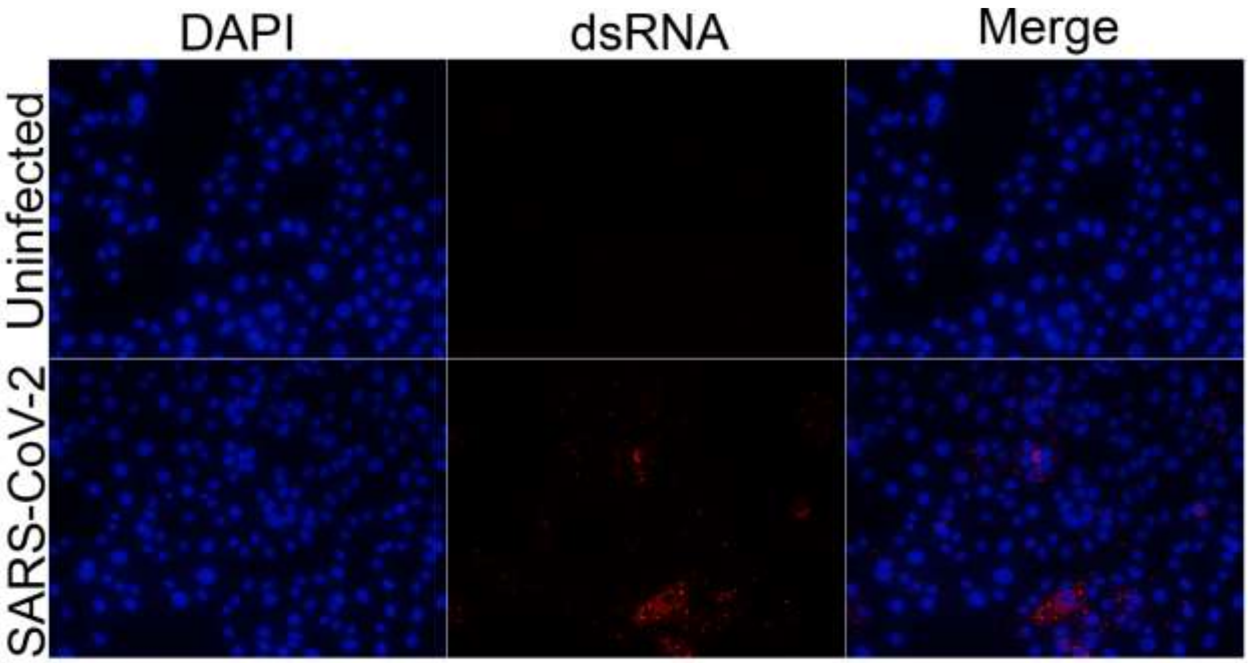

Figure S2. SARS-CoV-2 dsRNA in infected Vero cells. Vero cells infected at an MOI of 1 were fixed 24 hpi, stained with for dsRNA (J2, red) and for cell nuclei (DAPI, blue) and visualized with a total magnification of 200 times
